# Parent-reported emotion regulation in children with dyslexia and/or developmental language disorder

**DOI:** 10.3389/fpsyg.2026.1723695

**Published:** 2026-05-20

**Authors:** Taylor Jane Bryant, Erin Smolak, Dawna Duff, Suzanne Marie Adlof

**Affiliations:** 1 Department of Communication Science and Disorders, University of South Carolina, Columbia, SC, United States; 2 Division of Speech Language Pathology, Binghamton University, Binghamton, NY, United States

**Keywords:** developmental language disorder (DLD), dyslexia, emotion regulation, language, word reading

## Abstract

**Introduction:**

Recent research has found that children with dyslexia have increased mental health problems. However, little is known about their emotion regulation, an ability that is crucial to psychosocial and academic well-being. Often, developmental language disorder (DLD) co-occurs with dyslexia, and importantly, DLD is also associated with increased mental health problems as well as poor emotion regulation. Neither line of research on mental health has accounted for the co-occurrence of dyslexia and DLD; thus, it is possible that difficulties with emotion regulation in either population may be attributed to the co-occurrence of these problems rather than to either dyslexia or DLD occurring in isolation.

**Methods:**

We examined parent-reported emotion regulation in 278 second-grade children (M_age_ = 8;0 years; 124 female) with dyslexia, DLD, dyslexia+DLD or typical development (TD). Parents reported their children’s emotion regulation using the *Behavior Rating Inventory of Executive Function-2*, which asks parents to rate the general frequency and disruptiveness of behaviors thought to represent emotion regulation difficulties.

**Results:**

Results indicated that children with dyslexia were rated as having significantly worse emotion regulation difficulties on average compared to TD children, and that a higher proportion of children with dyslexia or dyslexia+DLD obtained ratings indicating elevated emotion regulation problems compared to TD children. Notably, we found no significant differences between children with DLD alone and TD children. When previous diagnoses of attention deficit/hyperactivity disorder were considered, patterns of estimates for dyslexia and dyslexia+DLD were similar but no longer statistically significant.

**Discussion:**

For children of this age and grade level, our findings suggest that emotion regulation difficulties are more associated with dyslexia than DLD, although we cannot elucidate whether the symptoms are due to emotional processing difficulties, insufficient executive control, or other causal factors. We discuss the need for further research on the emotional challenges of children with dyslexia and children with DLD that considers the co-occurrence of reading, language, and attention problems.

## Introduction

Dyslexia is a specific learning disability characterized by difficulties with reading at the word level (Siegel et al., 2025). Children with dyslexia, when compared to peers with typical reading, display increased rates of anxiety (Donolato et al., 2021; Donolato et al., 2024; Francis et al., 2019; Francis et al., 2022; Grills et al., 2023; McArthur et al., 2022; Robidoux et al., 2025; Vieira et al., 2024) and depressive symptoms (Donolato et al., 2021; Francis et al., 2019; Vieira et al., 2024). Because emotion regulation difficulties are a known risk factor for mental health difficulties across many populations (Cole et al., 2017; Fernandez et al., 2016; Tajik-Parvinchi et al., 2021) researchers such as Boyes et al. (2016) have called for research examining emotion regulation in children with dyslexia. However, initial studies comparing parent-reported emotion regulation in children with dyslexia to that of children with typical word reading ability have obtained mixed results (Gioia et al., 2002; Morte-Soriano et al., 2021). If children with dyslexia display emotion regulation problems, it would be concerning for multiple reasons. First, experiencing unwanted emotions, and being unable to control those emotions, can be painful (Solomon, 2002) and in some cases, it can account for poor social functioning (Bunford et al., 2014; Cleminshaw et al., 2020). Second, an inability to effectively regulate emotions could negatively affect learning and academic performance (Graziano et al., 2007). Given the small and inconclusive body of work on emotion regulation in children with dyslexia, and the potential of emotion regulation problems to impact multiple important domains of life, an examination of the emotion regulation of children with dyslexia is critical.

One variable that could potentially impact findings about emotion regulation in children with dyslexia is the co-occurrence of dyslexia with developmental language disorder (DLD). DLD is a life-long neurodevelopmental disorder that emerges in early childhood, and it is characterized by a significant difficulty learning, understanding, and using spoken language (McGregor, 2020). Dyslexia frequently co-occurs with DLD, although they are separable and distinct disorders (Adlof and Hogan, 2018; Catts et al., 2005; Snowling et al., 2020). Important to this discussion of emotion regulation, children with DLD have been found to display greater emotion regulation problems on average than children with typical development (Burnley et al., 2023; Forrest et al., 2020; Fujiki et al., 2002; Fujiki et al., 2004; Kuusisto et al., 2017; St. Clair et al., 2019; cf. Aguilera et al., 2021; van den Bedem et al., 2018) as well as increased risk for developing clinical levels of anxiety and depression (Botting et al., 2016a, b). However, neither the line of research on emotion regulation in children with dyslexia nor the line on children with DLD has examined their co-occurrence. Thus, it is currently unclear whether emotion regulation and mental health problems are related to dyslexia in particular, or whether they are related to having co-occurring word reading and language difficulties. To address this gap in the research, the current study examines parent-reported emotion regulation in children with dyslexia, children with DLD, children with co-occurring dyslexia and DLD (dyslexia+DLD), and children with typical development (TD).

Individuals vary in the intensity and speed of their emotional responses to events, or their emotional reactivity (Rothbart and Derryberry, 1982), as well as in their capacity to control their emotional responses. Further, the intensity and speed of an individual’s emotional responses may influence the success of an individual’s attempts to control or modulate them (Calkins and Perry, 2016). Thus, together, emotional reactivity and control jointly impact emotional displays and behaviors that are apparent to observers. Importantly, scientists vary in how they define and operationalize the term “emotion regulation”: some consider emotion regulation to include reactive emotional responding to stimuli in addition to executive/top-down efforts to control emotional responses (e.g., Crowell, 2021; Fox and Calkins, 2003), whereas others consider emotion regulation to consist of the regulatory component alone (Gross, 2015). Because our study examines parent ratings of children’s displays of emotional behavior, we do not differentiate between emotional reactivity or regulation. The visible behaviors that parents are asked to consider and rate are the behavioral outputs of both the reactive and regulatory components of emotion regulation working together. Therefore, for the purposes of the current study, we consider emotion regulation to be the ability to respond to emotional stimuli in alignment with one’s individual goals, changing or maintaining emotional states as needed through the engagement of cognitive and behavioral processes (Calkins, 1994; Fox and Calkins, 2003).

Effective emotion regulation allows an individual to flexibly transition from one emotion-eliciting situation to another; it is theorized to be implicated in everyday situations through its support of goal-directed behavior, whether those goals are social (Porges, 2003), academic (Harley et al., 2019), achievement-related (Harley et al., 2019), or purely hedonic (Gross, 2015). Children’s emotion regulation is frequently assessed through self-report or third-party questionnaires (see Mazefsky et al., 2021 for a review), often as part of larger standardized inventories of executive functions (e.g., Barkley, 2012; Gioia et al., 2000; Gioia et al., 2015; Naglieri and Goldstein, 2013). Third-party questionnaires ask parents or teachers rate the severity and/or frequency of behaviors they observe, such as emotional outbursts or instances of upset. Empirically, emotion regulation has been shown to play a role in an individual’s formation and maintenance of friendships (English et al., 2012; Ricciardi et al., 2022), emotional engagement at school (De Neve et al., 2023), and school-related well-being vs. burnout (Beaumont et al., 2023; Seibert et al., 2017). Indeed, as early as kindergarten, emotion regulation has longitudinally predicted social skills (Blair et al., 2015) and academic success (Graziano et al., 2007). Poor emotion regulation has been associated with internalizing (e.g., anxiety and depression) and externalizing (e.g., conduct) problems in children and youth (Compas et al., 2017). Importantly, meta-analyses have shown that across many studies, interventions have been successful in improving emotion regulation in children and adolescents (Espenes et al., 2024; Moltrecht et al., 2020), and evidence suggests the potential for emotion regulation interventions to positively impact academic performance (Thierry et al., 2016; Ramirez and Beilock, 2011; Verhaeghen, 2023).

Children with dyslexia primarily struggle with word reading, decoding, and spelling as demonstrated by low accuracy and/or fluency on these tasks (Adlof and Hogan, 2018; Catts et al., 2024; International Dyslexia Association, 2025; Siegel et al., 2025). Children with dyslexia also experience increased anxiety and/or depression relative to their peers with typical reading (Donolato et al., 2021; Donolato et al., 2024; Francis et al., 2019; Francis et al., 2022; Grills et al., 2023; McArthur et al., 2022; Robidoux et al., 2025; Vieira et al., 2024). A line of qualitative research on children with dyslexia has identified patterns of intense emotional responses with negative valences occurring in childhood (e.g., frustration, anxiety, shame, or despair): adults with dyslexia have described childhood academic experiences as being emotionally painful (Carawan et al., 2016; Nalavany et al., 2018). Recently, Wilmot et al. (2023) used reflexive thematic analysis to examine interviews of children with dyslexia; in these interviews, children with dyslexia reported school-related worry, stress, and embarrassment, and their mothers described their children having academics-related meltdowns, school refusals, and homework resistance. Importantly, having dyslexia can be apparent to others, such as peers, parents, and teachers—being asked to perform reading tasks aloud or being grouped according to reading ability within a classroom may bring a child’s attention to their dyslexia and induce worries about how peers, parents, and teachers perceive their word reading difficulties (see Leitão et al., 2017; Wilmot et al., 2023). Hence, it could be that the accumulation of these difficult learning and school-related experiences involving heightened negative emotions increases the risk of developing emotion regulation problems.

It is possible that emotion regulation difficulties are a risk factor for mental health symptom development in children with dyslexia (Boyes et al., 2016). In an initial study of casefile data of children with diagnosed dyslexia, Boyes et al. (2020) found that parent-reported emotion regulation problems were predictive of the children with dyslexia who later developed internalizing or externalizing mental health symptoms. However, their study lacked a comparison group, and thus, could not determine whether emotion regulation abilities were related to the diagnosis of dyslexia, or whether emotion regulation could moderate or mediate a potential relationship between dyslexia and mental health symptomatology. Furthermore, extant research comparing emotion regulation in children with dyslexia to TD children has found mixed results. Two studies compared the emotion regulation of children with dyslexia to children with TD, using questionnaires completed by parents and/or teachers to evaluate emotion regulation, specifically the Behavior Rating Inventory of Executive Function (Gioia et al., 2000) and the Spanish version of its revision (Gioia et al., 2017), and they yielded conflicting results. Morte-Soriano et al. (2021) found that a sample of 9–15 year-old children with dyslexia from Spain had significantly worse emotion regulation than children with TD based on parent and teacher report, but Gioia et al. (2002) found no significant differences in parent-reported emotion regulation between these groups in their sample from the United States. However, Gioia et al. (2002) provided little information about the ascertainment of their sample, and on average, their dyslexia group (M _age_ = 8.8) was roughly 2 years younger than their control group (M_age_ = 10.9). Given the relatively few studies focusing on emotion regulation in children with dyslexia and possible differences in their study design, further examination is needed to conclude whether emotion regulation problems are present in children with dyslexia to a greater degree than seen in TD children.

Many children with dyslexia have co-occurring language weaknesses; in fact, past studies of the co-occurrence of dyslexia and DLD have found rates of overlap varying between 17 and 71% (Adlof and Hogan, 2018; Catts et al., 2005; McArthur et al., 2000). It has been argued that language supports humans in making sense of emotions (Lindquist et al., 2015). The ability to label emotions may assist children in developing emotion understanding and recognition in oneself and others by enabling them to use categorical perception (Eisenberg et al., 2005; Feldman Barrett, 2017). Furthermore, language enables children to self-regulate through private or inner speech (Winsler et al., 2009) and supports children in communicating their needs, which may itself be a form of emotion regulation (Cole et al., 2010). Thus, it is theorized that children with language difficulties, such as those with DLD, may then struggle with emotion recognition, understanding, and regulation (Eisenberg et al., 2005). Indeed, children with DLD have been found to have poorer emotion regulation in preschool and elementary grades than their peers with typical language (Burnley et al., 2023; Forrest et al., 2020; Fujiki et al., 2002; Fujiki et al., 2004; Kuusisto et al., 2017; St. Clair et al., 2019). However, no significant differences between school-age children with DLD and TD children in their parent-reported emotion regulation were found in a sample from Spain (Aguilera et al., 2021). Another study found that children with DLD do not report using significantly different types of emotion regulation strategies differently from children with TD (van den Bedem et al., 2018). Notably, this literature is small, and definitive conclusions about emotion regulation in children with DLD cannot currently be drawn, though the majority of studies have found a connection between emotion regulation difficulties and DLD.

Importantly, comparing emotion regulation in groups of children with dyslexia, with DLD, and with co-occurring dyslexia+DLD to TD children may help identify which factors may be most critical to consider when examining emotion regulation. This comparison has yet to be made in research on emotion regulation difficulties or internalizing or externalizing mental health problems more broadly. For example, a recent meta-analysis conducted by Donolato et al. (2021) found that across studies, in comparison to TD children, children with reading disorders and children with language disorders both exhibit increased internalizing and externalizing symptoms. Of further interest, they found that the group difference in internalizing symptoms was moderated by the primary disorder type. Children with language disorders displayed more internalizing problems than children with reading disorders; thus, it is possible that internalizing difficulties are more closely tied to having a language disorder. However, a critical gap remains: none of the studies included in this meta-analysis accounted for the co-occurrence of reading and language difficulties in their examinations of internalizing and externalizing problems. Having both dyslexia and DLD in childhood may confer additional risk for emotion regulation difficulties. That is, an additive effect on emotional functioning may be seen for children who experience both difficulties with word reading and with language; having multiple difficulties in childhood often increases likelihood of poor outcomes in adolescence and adulthood (Appleyard et al., 2005; Evans et al., 2013; Hayiou-Thomas et al., 2021). In theory, these children with dyslexia+DLD would experience academic struggles associated with word reading as well as difficulty recognizing and communicating their emotions. Indeed, children with co-occurring dyslexia+DLD have been found to perform significantly lower in school than children with dyslexia, children with DLD, and children with TD (Duff et al., 2023); it is possible that these children encounter greater educational adversity than their counterparts with a single learning difficulty and therefore, experience greater risk for emotion regulation problems.

On the other hand, emotion regulation difficulties may underly the diagnoses of dyslexia and DLD as part of a broader difficulty with executive functioning seen across neurodevelopmental disorders. Executive functioning is related to emotion regulation in that it consists of higher-order, top-down cognitive processes that permit humans to direct their own thoughts, actions, and emotions rather than responding automatically to environmental stimuli (Diamond, 2013; Zelazo, 2020). Attention deficit/hyperactivity disorder, or ADHD, is a difficulty with executive functioning and it frequently co-occurs with both dyslexia and DLD (Gayán et al., 2005; Gilger et al., 1992; Loo et al., 2004; Redmond, 2020; Willcutt and Pennington, 2000; Yew and O’Kearney, 2013). Alongside poorer general self-regulation, most children with ADHD experience increased emotional lability, which also contributes to emotion regulation difficulty (Christiansen et al., 2019; Graziano and Garcia, 2016). Importantly, England-Mason (2020) highlighted the role of emotion regulation as a transdiagnostic feature in ADHD and autism spectrum disorder. She argued for further study of emotion regulation processes in children with communication disorders, alongside other neurodevelopmental disorders, to determine whether emotion regulation difficulties may cut across neurodevelopmental disorders as a shared characteristic or mechanism.

To our knowledge, no studies of emotion regulation have considered the co-occurrence of dyslexia and DLD, which could obscure understanding of the mechanisms for development of poor emotion regulation in these populations. The current study represents a first step toward filling this gap by comparing emotion regulation, as measured by parent report on the Behavior Rating Inventory of Executive Function-2 (BRIEF-2) questionnaire, in children with dyslexia, with DLD, and with dyslexia+DLD in comparison to children with TD. Data were collected as part of a larger study of spoken word-learning involving second-grade children from these groups.

Our study investigates the following research questions:

1. Do children with dyslexia, DLD, and dyslexia+DLD differ from children with TD in emotion regulation as measured by the Emotion Regulation Index of the BRIEF-2, Parent-Report?
2. Do children with dyslexia, DLD, and dyslexia+DLD show greater risk of elevated emotion regulation problems as compared to children with TD?

Given that past research focused on dyslexia and DLD separately, we predicted that the children in the dyslexia+DLD group would be rated by their parents as demonstrating significantly poorer emotion regulation than children with TD. We also predicted that children in the dyslexia+DLD group would have a significantly higher risk of elevated emotion regulation problems than children with TD. However, we made no hypotheses about whether dyslexia or DLD alone would be associated with worse emotion regulation or higher risk of elevated emotion regulation difficulties. Because ADHD commonly co-occurs with dyslexia (Gayán et al., 2005; Gilger et al., 1992; Loo et al., 2004; Willcutt and Pennington, 2000) and DLD (Redmond, 2020), and because most children with ADHD experience deficits in emotional reactivity and/or emotion regulation (Graziano and Garcia, 2016), we performed sensitivity analyses for each research question, taking into consideration parent reports of prior ADHD diagnoses.

## Method

The current study utilized data collected as part of a larger project conducted at the University of South Carolina and the University of Pennsylvania, which focused on word-learning in children with dyslexia or DLD. Study procedures were approved by the Institutional Review Board at each university. We recruited participants over a period of five academic years (2019–2024) using multiple methods, which included targeted advertisements that were delivered online through recruitment registries, society e-mail listservs, and social media, and through referrals from speech-language pathologists and related professionals. Parents gave signed informed consent for their child’s participation in the study prior to data collection. They then completed an intake questionnaire, which requested demographic and developmental history information.

### Participants

Participants were 278 second-grade children (M_age_ = 8;0 years; 124 female) from the larger project whose parents had completed the BRIEF-2 Parent Report. According to parent responses on the study intake questionnaire, all participants spoke English as their primary language, and none had a medical or developmental history that would interfere with speech or language development (e.g., hearing impairment, autism spectrum disorder, genetic syndromes, or traumatic brain injury). We retained 267 participants for our analytic sample based on validity criteria described in the BRIEF-2 manual (see Supplementary materials); the demographics reported below reflect our analytic sample.

Consistent with the inclusion criteria for our larger project and other research on dyslexia and DLD (e.g., Duff et al., 2023; Grills et al., 2023; McGregor et al., 2023; Hancock et al., 2025), we did not exclude children with prior diagnoses of ADHD from any group. ADHD occurs in children who do not have word reading or language difficulties (DuPaul et al., 2013), though it is known to frequently co-occur with dyslexia (Sexton et al., 2012) and DLD (Redmond, 2020; Yew and O’Kearney, 2013), and we retained these children to support the generalizability of our findings to the broader population. However, we observed that our impairment subgroups differed in the percentage of children who had a prior diagnosis of ADHD as indicated by parents on the intake questionnaire (TD: 3.7%, Dyslexia: 22.6%, DLD: 9.1%, Dyslexia+DLD: 28.6%). Given that an estimated 50–75% of children with ADHD show symptoms aligning with emotion regulation difficulty (Drechsler et al., 2020), we elected to conduct sensitivity analyses considering ADHD status.

Approximately 21.8% of participants were from South Carolina and 43.2% of participants were from Pennsylvania; the remainder of participants were distributed throughout the United States. The racial identities of participants as reported by parents were: 1.8% Asian, 7.5% Black, 6.0% multiracial, and 80.9% White, 2.3% Other, 1.5% Unreported. Parents reported 92% of participants were not Hispanic/Latino and 4.4% were Hispanic/Latino (3.5% Unreported).

### Word reading and general language measures and procedures

Participants were classified into dyslexia, DLD, dyslexia+DLD, and TD groups based on their performance on norm-referenced tests of word reading and language and parent information provided on the parent questionnaire. The test battery was administered over several sessions held in a quiet room in the school, in the investigator’s lab, or online via teleconferencing.

Word reading performance was measured with the Word Identification and Word Attack subtests of the Woodcock Reading Mastery Test-III (WRMT-III; Woodcock, 2011). The WRMT-III (and previous versions) are commonly used in research and clinical practice to identify children with dyslexia (e.g., Hancock et al., 2025; Marks et al., 2024). The subtests measure children’s accuracy of reading real words (Word Identification) and pseudowords (Word Attack) of increasing difficulty when they are presented in isolation. Scores from the two subtests combine to derive the Basic Skills composite score, which according to the test manual shows good reliability for the age ranges represented in our sample (split half = 0.96 to 0.97). Children who earned a standard score of 85 or lower were classified as having dyslexia (e.g., Catts et al., 2005; Joanisse et al., 2000; Siegel, 2008).

Language ability was measured using the Core Language Index score from the Comprehensive Evaluation of Language Fundamentals–Fifth Edition (CELF-5; Wiig et al., 2013), which is commonly used in the clinical diagnosis of language disorders in children (Betz et al., 2013; Denman et al., 2017). This index score quantifies a student’s general language performance, and it is derived from four subtests assessing different aspects of linguistic expression and comprehension (e.g., syntax, morphology, and semantics). For the age ranges within our sample, the internal consistency of the Core Language Index score is excellent (0.95 to 0.97). Children whose index score was 85 or lower were classified as having DLD. This cut score is consistent with prior research (Hancock et al., 2025; Hannig Russell and Redmond, 2025; Werfel and Krimm, 2017), and according to the test manual, it provides 100% sensitivity and 91% specificity for identifying children with language impairment in ages 7;0–9;11. Participants additionally completed the Diagnostic Evaluation of Language Variation Screening Test (DELV-ST; Seymour et al., 2003), and we used this data to ensure that children whose spoken dialect varied from General American English were appropriately classified (see Supplementary materials).

Children scoring > = the 25th percentile (SS > = 90) on both the Basic Skills Cluster of the WRMT-III and the Core Language Score of the CELF-5 were classified as having TD. Children who were reported by their parents to have been previously diagnosed with dyslexia or DLD were excluded from this group, as were children who were reported by their parents to have received small group or individual reading intervention. If children scored ≤ the 16th percentile (SS ≤ 85) on the Basic Skills Cluster of the WRMT-III, but > the 16^th^ percentile on the Core Language Score of the CELF-5 (SS > 85), they were classified as having dyslexia. If children scored > the 16th percentile (SS > 85) on the Basic Skills Cluster of the WRMT-III, but ≤ the 16^th^ percentile on the Core Language Score of the CELF-5 (SS ≤ 85), they were classified as having DLD. Children scoring ≤ the 16th percentile (SS ≤ 85) on both the Basic Skills Cluster of the WRMT-III and the Core Language Score of the CELF-5 were classified as dyslexia+DLD.

Table 1 contains descriptive statistics for each group on the subgrouping assessments.

**Table 1:** Sample performance on general language and word reading measures.

Group	WRMT-III Basic Skills Cluster		CELF-5 Core Language Score	
	*M*	*SD*	*M*	*SD*
TD (*n* = 164)	108.75	11.30	107.92	11.09
Dyslexia (*n* = 53)	77.94	5.25	95.98	6.70
DLD (*n* = 22)	94.90	6.21	79.77	4.03
Dyslexia+DLD (*n* = 28)	74.96	6.69	77.86	5.85

WRMT-III Basic Skills Cluster and CELF-5 Core Language scores were used for group classification. Group-level means and standard deviations are provided for descriptive purposes.

### Emotion regulation measures and procedures

Emotion regulation was assessed using parent report via the Emotion Regulation Index (ERI) of the BRIEF-2. The BRIEF-2 consists of 63 items within 9 clinical scales, with its items intermixed; it is designed to assess the everyday executive functioning of children aged 5 to 18 through informant reports on the frequency of specific behaviors within situations in natural environments (Gioia et al., 2015). BRIEF-2’s ERI is a composite of two clinical scales, and it provides information on a child’s ability to regulate emotional responses generally (the Emotional Control scale; 8 items) and in the context of changing circumstances or demands (the Shift scale; 8 items). Using general population and clinical samples, Gioia et al. (2015) performed a confirmatory factor analysis which supported a three-factor structure of the BRIEF-2 and provides evidence for the separation of the emotion regulation dimension from the behavioral and cognitive regulation dimensions; this three-factor structure has been replicated in other clinical samples (Halvorsen et al., 2019). In these studies, the factor loadings, factor correlations, and model fit indices supported the inclusion of emotion regulation as measured by the ERI in their measure of global executive functioning. Gioia et al. (2015) also determined that the ERI exhibits moderate correlations with Child Behavior Checklist (Achenbach, 1991) scales and composites measuring emotional functioning, and strong correlations with Behavior Assessment System for Children, Second Edition (Reynolds and Kamphaus, 2004) scales and composites measuring emotional functioning. These correlations provide convergent evidence for the validity of the BRIEF-2 ERI in assessing emotion regulation.

For each item, parents rated behaviors as occurring never (corresponding to 1 point), sometimes (corresponding to 2 points), or often (corresponding to 3 points) within the last 6 months; the points were summed to obtain raw scores for individual scales. The BRIEF-2 provides T-scores that are normed based on age and gender for individual scales as well as its indexes; for all scales and composites, higher scores indicate greater parent-reported difficulty. Parent forms for the Shift and Emotional Control scales and the ERI have high internal consistency, as reported by the test manual (Shift: *α* = 0.84; Emotional Control: α = 0.91; ERI: α = 0.92) and as seen in our sample (Shift: α = 0.86; Emotional Control: α = 0.91; ERI: α = 0.92). We followed recommendations of the BRIEF-2 manual to ensure the validity of parent responses. Due to inconsistent endorsement of related items, and endorsement of items that were highly unlikely to be true based on the ages of children in the sample, 11 participants were excluded from the analytic sample (see Supplementary materials for more information).

### Analytic approach

Multiple analytic procedures were used to answer our research questions. We examined differences between the group’s mean levels of emotion regulation difficulty, as measured by BRIEF-2 ERI Score, by estimating regression models with group as a dummy-coded variable. To address our first research question, our first model, Model A, included only the group explanatory variable. However, given the observed differences in the proportion of students with ADHD across groups, we also ran a second model, Model B, which included previous diagnosis of ADHD as an additional dummy-coded predictor variable. Assumptions were checked for both regression models using the variance inflation factor, the Durbin–Watson statistic and visual examination of Q–Q (quantile–quantile) plots. Both models met assumptions. We assessed model fit with a likelihood ratio test and calculated standardized coefficients to serve as an effect size of the difference in emotion regulation between each group and the TD reference group.

Our second research question focused on group differences in risk of elevated emotion regulation problems. According to the BRIEF-2 test manual, scores 60–64 on the BRIEF-2 are considered mildly elevated, with scores of 65–70 considered potentially clinically elevated and scores ≥ 70 considered clinically elevated (Gioia et al., 2015); we considered scores ≥ 60 to be elevated. We planned to use three Pearson’s chi-square tests (Fisher, 1992; Pearson, 1900) to test our null hypotheses. However, one cell’s frequency count was too low to meet the standard assumption for chi-squre (i.e, frequency count was < 5; see Results). Thus, we used Fisher’s exact test to test for a difference in the TD and DLD groups’ rates reaching thresholds indicating elevated difficulty. All other assumptions were met for our Pearson’s chi-square tests and the Fisher’s exact test. We also performed a Pearson’s chi-square test and two Fisher’s exact tests focusing only on children without a prior diagnosis of ADHD (TD: *n =* 158; Dyslexia: *n* = 41; DLD: *n* = 20; Dyslexia+DLD: *n* = 20) to determine whether the pattern of results from the first tests was maintained. We calculated odds ratios to serve as an effect size of group differences in proportions. All analyses were conducted in R (Version 4.3.1; R Core Team, 2023) using multiple packages for data manipulation, analysis, and visualization (Behrendt, 2023; Komsta and Novomestky, 2022; Warnes et al., 2022; Wickham, 2016; Wickham et al., 2019).

**Table 2:** Contingency and proportion table displaying counts and proportions of groups with elevated and typical emotion regulation scores.

Emotion Regulation Index score category	Previous diagnoses of ADHD included		Previous diagnoses of ADHD excluded	
	TD	Dyslexia	TD	Dyslexia
Elevated	36 (0.22)	19 (0.36)	33 (0.21)	12 (0.29)
Typical	128 (0.78)	34 (0.64)	125 (0.79)	29 (0.71)
Total	164	53	158	41
		DLD		DLD
Elevated		3 (0.14)		2 (0.10)
Typical		19 (0.86)		18 (0.90)
Total		22		20
		Dyslexia+DLD		Dyslexia+DLD
Elevated		11 (0.39)		6 (0.30)
Typical		17 (0.61)		14 (0.70)
Total		28		20

## Results

The first research question asked whether children with dyslexia, DLD, and dyslexia+DLD differed from children with TD in their emotion regulation. Mean T-scores and standard deviations for each group on the Emotional Control scale, the Shift scale, and the ERI are reported in Table 3 alongside the other BRIEF-2 composites.

**Table 3:** Descriptive statistics for BRIEF-2 individual scale and composite T-scores by group.

Individual Scale or Composite Name	TD (*n* = 164)	Dyslexia (*n* = 53)	DLD (*n* = 22)	Dyslexia + DLD (*n* = 28)
Shift	50.20 (10.27)	54.62 (12.84)	48.86 (12.84)	56.39 (14.15)
Emotional control	52.54 (11.10)	54.72 (11.92)	48.41 (9.17)	53.39 (10.40)
Emotion Regulation Index	51.26 (9.95)	55.15 (12.27)	48.50 (9.78)	55.29 (12.41)

Numbers reported in the table correspond to the group’s mean T-Score with the standard deviation for that group reported in parentheses.

**Figure 1:**
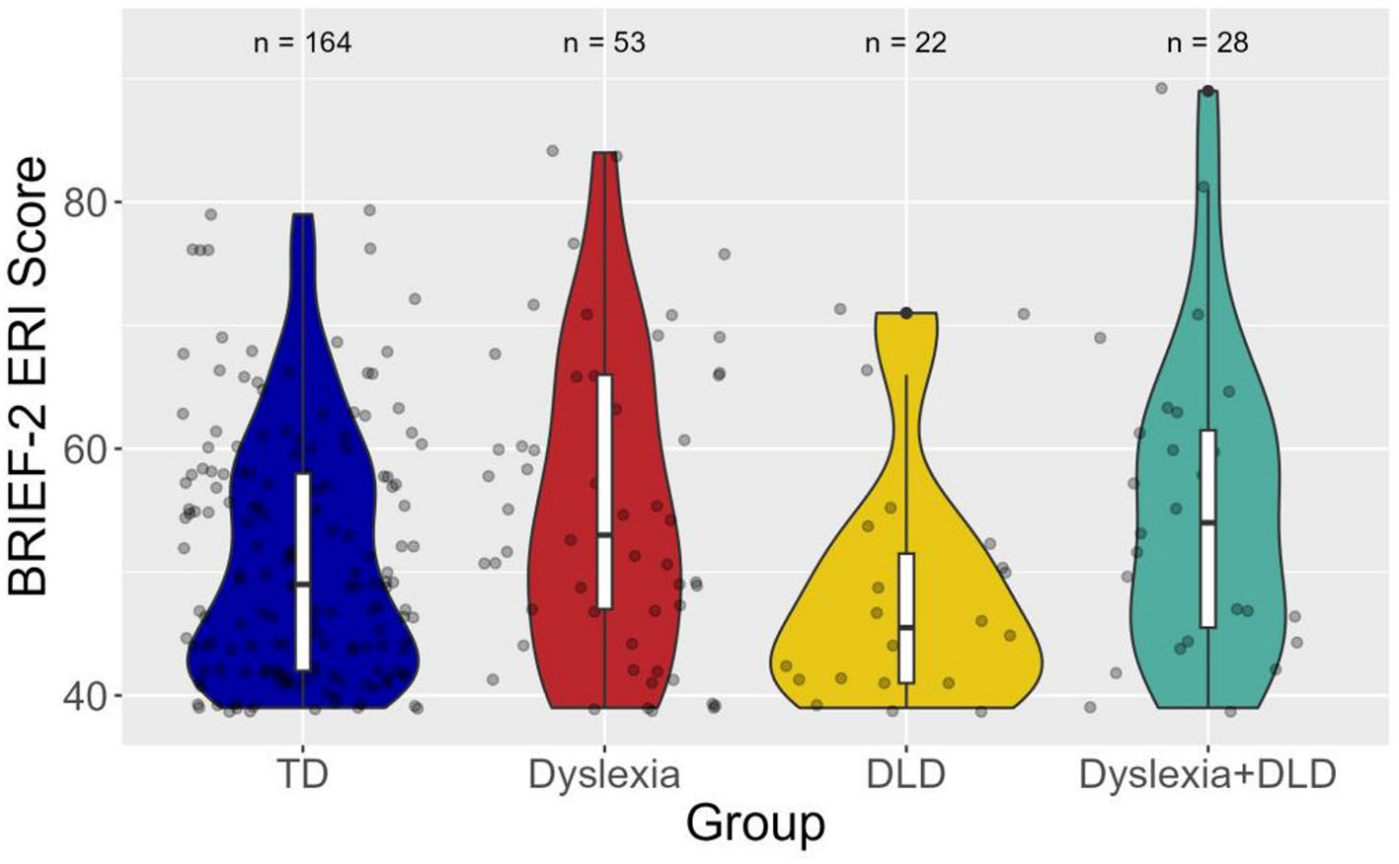
Violin plot displaying BRIEF-2 Emotion Regulation Index T-score distributions by group. A higher T-score indicates greater parent-reported difficulties with emotion regulation. Scores ≥ 60 are considered elevated.

Figure 1 shows a violin plot of the distributions of ERI scores by group. As can be seen in the figure, the distributions of scores observed in the TD, dyslexia, and dyslexia+DLD groups included higher scores than were observed in the DLD group. Table 4 reports the results of Model A, which included only the group explanatory variable, and the results of Model B, which added previous diagnosis of ADHD as a covariate.

**Table 4:** Multiple regression models testing for group differences in emotion regulation.

Model	*F*	*df*	*p*	Predictors	Estimate	Std. error	*β*	*t*	*p*
Model A	3.42	3, 263	0.017*	Intercept	51.26	0.84	-	61.31	<0.001*
				Group					
				Dyslexia	3.90	1.69	0.14	2.30	0.022*
				DLD	−2.76	2.43	−0.07	−1.13	0.258
				Dyslexia+DLD	4.03	2.19	0.11	1.84	0.067
Model B	6.09	4, 262	<0.001*	Intercept	50.96	0.82	-	62.10	<0.001*
				Group					
				Dyslexia	2.35	1.70	0.09	1.381	0.168
				DLD	−3.20	2.38	−0.08	−1.35	0.180
				Dyslexia+DLD	2.01	2.21	0.06	0.91	0.364
				ADHD	8.12	2.20	0.23	3.69	<0.001*

The TD group is the referent for the group effect. *indicates statistical significance given threshold of *p* < .05.

Results of Model A indicated that there was a significant effect of group on ERI scores (*F* = 3.42, *p* < 0.001).^1^ The estimated mean score for children with dyslexia was 3.9 points higher (i.e., worse) than children with TD. This difference in means was small but statistically significant (*β* = 0.14, *p* = 0.022). The average ERI score for children with dyslexia+DLD was estimated to be 4.03 points higher than the TD group, but this difference was statistically marginal *(β* = 0.11, *p* = 0.067). In contrast, there was an estimated −2.76 point difference between children with DLD and children with TD in average ERI scores, suggesting that children with DLD had better emotion regulation than children with TD, but this difference was not significant *(β* = −0.07, *p* = 0.258).

ADHD status was included as an additional predictor in Model B, which was also significant *(F* = 4.69, *p* < 0.001). However, in this model the estimated differences between ERI scores for the dyslexia, DLD, and dyslexia+DLD groups compared to the TD group were smaller and no longer statistically significant (see Table 4). The estimated ERI score for children with a previous diagnosis of ADHD was 8.12 points higher than children without ADHD and statistically significant (*β* = 2.20, *p* < 0.001), reflecting significantly worse emotion regulation in children with a prior diagnosis of ADHD than for those with no prior diagnosis of ADHD.

Our second research question asked whether children with dyslexia, with DLD, and with dyslexia+DLD have greater risk of receiving ratings of elevated emotion regulation (ER) problems than children with TD. Table 2 displays frequencies and proportions of each group with elevated and typical ERI scores. Results for the second research question followed a similar pattern to those of the regression analyses. A significantly larger proportion of children with dyslexia received ratings indicating elevated emotion regulation problems compared to the proportion of children with TD [*Χ*^2^(1) = 4.09, *p* = 0.043]; the odds ratio was 1.98. Similarly, a significantly larger proportion of children with dyslexia+DLD received ratings indicating elevated emotion regulation problems compared to that observed for children with TD [*Χ*^2^(1) = 3.89, *p* = 0.049]; the odds ratio was 2.29. Although a smaller proportion of children with DLD received ratings indicating elevated emotion regulation problems in comparison to the proportion of children with TD, this group difference was not significant (*p* = 0.567, odds ratio = 0.56). When children with prior diagnoses of ADHD were excluded, the pattern of results obtained was similar to those observed in our follow-up regression analysis accounting for ADHD status. Larger proportions of elevated emotion regulation problems were still seen in the dyslexia and dyslexia+DLD groups as compared to the proportion observed for the TD group, but a smaller proportion of elevated emotion regulation problems was seen in the DLD group as compared to the TD group. However, none of these differences between groups in rates of elevated emotion regulation problems reached statistical significance [dyslexia and TD: *Χ*^2^(1) = 1.31, *p* = 0.253, odds ratio = 1.56; dyslexia+DLD and TD: *p* = 0.391, odds ratio = 1.62; DLD and TD: *p* = 0.577, odds ratio = 0.56], though it should be noted that removing children from the sample reduced the statistical power.

## Discussion

We investigated whether second-grade children (mean age = 8 years) with dyslexia, with DLD, and with dyslexia+DLD differ from children with TD in emotion regulation as measured by the reports of their parents on the BRIEF-2. To date, two separate bodies of research have suggested that poor emotion regulation may exist in children with dyslexia and children with DLD. Researchers have hypothesized that frequent negative experiences while reading, and awareness and fear of how they might be perceived by peers and teachers, may contribute to emotion regulation difficulty in children with dyslexia (e.g., Leitao et al., 2017), whereas children with DLD have been shown to have impaired ability to recognize, communicate, and internally mediate emotions through language (e.g., Bahn et al., 2021; Rieffe and Wiefferink, 2017; Spackman et al., 2006), which has been hypothesized to contribute to their poor emotion regulation. However, because past studies of emotion regulation in children with dyslexia or DLD have not accounted for co-occurring dyslexia and DLD (Burnley et al., 2023; Forrest et al., 2020; Fujiki et al., 2002; Fujiki et al., 2004; Gioia et al., 2002; Kuusisto et al., 2017; Morte-Soriano et al., 2021; St. Clair et al., 2019), we cannot determine the extent to which results reflect the emotion regulation of children with co-occurring difficulties. The current study provides initial results toward filling this research gap.

We used two analytic approaches to address our research questions. First, we examined overall group differences in mean emotion regulation abilities as measured by scores on the BRIEF-2 parent scales. We found significant differences between children with dyslexia and TD children, such that children with dyslexia were determined to have worse emotion regulation than children with TD. Means for children with dyslexia+DLD were also determined to be worse than for TD children, although the difference was beyond the threshold for statistical significance (*p* = 0.066). In contrast, no significant differences were found between the DLD group and the TD group. Second, we examined whether there were differences in the proportion of children from each group who showed risk for elevated emotion regulation difficulties. Importantly, most children with dyslexia, DLD, and dyslexia+DLD had adequate emotion regulation, as rated by their parents. However, consistent with the previous analyses, we found significantly higher proportions of children with elevated emotion regulation difficulties in the dyslexia and in the dyslexia+DLD group compared to the TD group; children in the dyslexia and dyslexia+DLD groups were approximately twice as likely as TD children to receive a rating indicating elevated emotion regulation difficulties. In contrast, the proportion of children in the DLD group with elevated emotion regulation difficulties was lower than, and not significantly different from, the TD group. Our results also suggest that it is important to pay attention to the co-occurrence of ADHD. With previous diagnoses of ADHD was included in the model, the estimates for the dyslexia, dyslexia+DLD, and DLD groups were not statistically significant. Likewise, when children with ADHD were removed from the sample, we found no statistically significant differences between rates of elevated parent-reported emotion regulation problems. This aligns with other recent work: Vieira et al. (2025) found that children with co-occurring word reading and math difficulties were more likely to show internalizing problems than children with typical reading and math development, and that attention likely contributes to this relationship.

In the past, dyslexia has been defined as word reading difficulties that are “unexpected” given an individual’s cognitive ability and exposure to reading instruction (Lyon et al., 2003; U.S. Office of Education, 1977), although the operationalization of this criterion has varied. The current study applied a performance-based criterion commensurate with some past research on dyslexia (Catts et al., 2005; Joanisse et al., 2000; Siegel, 2008) as well as contemporary definitions of dyslexia (Catts et al., 2024; International Dyslexia Association, 2025; Siegel et al., 2025), and it was unable to consider the quality of reading instruction that children had received. Therefore, it is possible that our findings may reflect a broader and more heterogeneous population of “struggling readers” in which ADHD plays a central role in development of emotion regulation problems. However, it is also possible that the consideration and receipt of a diagnosis of ADHD is incited by academic struggle and/or emotion regulation problems. Evidence suggests greater public awareness of ADHD than DLD or dyslexia (Bishop, 2010; de Lemos et al., 2022; Thordardottir and Topbaş, 2021), and ADHD can be diagnosed by pediatricians and primary care physicians. In contrast, children meeting criteria for dyslexia and/or DLD often remain unidentified (Christopulos and Kean, 2020; Hendricks et al., 2019; McGregor, 2020; Norbury et al., 2016; Truckenmiller, 2024) and even if identified for services, may be served under other diagnostic categories (including but not limited to “speech-language impairment; receptive-expressive language disorder; learning disability in reading; reading disorder; developmental learning disorder,” among others; Reilly et al., 2014; Sadusky et al., 2022). Therefore, the parents of children with dyslexia and/or emotion regulation difficulties may have voiced general concerns for emotion regulation and academic performance to their child’s doctor, and because ADHD was a better-known and more readily available diagnosis, this was the diagnosis that was given.

Although we did not test for significant differences between the dyslexia, DLD, and dyslexia+DLD groups, descriptive information may offer some insight. Mean ERI scores and proportions of elevated risk were highly similar for the two groups with dyslexia, and they were higher than the TD group. Such results suggest that, at least for children of this age/grade level, having DLD in addition to dyslexia did not increase mean levels of emotion regulation difficulties or the proportion of children with elevated levels. That is, we did not find an additive effect on emotional functioning in children who experience both difficulties with word reading and with language. This result is similar to McArthur et al.’s (2023) finding that 6-12-year-old children with combined dyslexia and language difficulties did not differ from children with dyslexia alone in risk for elevated anxiety symptoms. Additionally, we found that children with DLD alone did not significantly differ from TD children in mean ERI ratings or proportion of elevated risk; indeed, their means and proportions were lower than the TD group. Previous work in children with DLD has included arguments that DLD may be a mechanism for development of poor emotion regulation, given that DLD may impede children’s ability to recognize, name, and communicate about their emotions (Bahn et al., 2021; Rieffe and Wiefferink, 2017; Spackman et al., 2006). However, our study’s results suggest that for second grade students, emotion regulation difficulties are more highly associated with dyslexia than DLD. Though we cannot ascertain the etiology of emotion regulation difficulties in either children with dyslexia or DLD from this initial, cross-sectional study, we consider some possible explanations for our results here.

First, it is possible that for second-grade students, word reading difficulties have a greater impact on perceptions of academic ability than language difficulties do (cf. Duff et al., 2023). It is at this stage of development that children begin evaluating their own abilities relative to their peers and where their self-evaluations are particularly influenced by negative feedback (Ruble et al., 1980). Given that formal reading instruction generally begins in elementary school, children with dyslexia may be unused to having these problems, which could increase the likelihood of emotional displays. Thus, dyslexia may be more salient to children and their families (e.g., Chan et al., 2022; Nation et al., 2004) and may more frequently present opportunities for emotional challenge, leading parents to report frequent emotion regulation problems in their children with dyslexia. In contrast, language delays in children with DLD are measurable from toddlerhood even though they are often not clinically identified (Sansavini et al., 2021). By second grade, children with DLD may not be experiencing daily communication demands that differ from what they have already encountered from a young age. Thus, they may have habituated to that level of difficulty. Further, if children with DLD display word reading abilities that are commensurate with age and grade expectations, they may not necessarily be experiencing the same emotional challenge in their school environment as children with dyslexia at this timepoint. Indeed, McGregor et al. (2023) found that in a sample of first-grade children with DLD, 37% met criteria for an academic disability, 26% displayed sub-clinical academic weaknesses, and another 37% did not present with any academic weaknesses; they found that children with DLD who presented with multiple risk factors were more likely to present with limits on daily functioning (academic and otherwise) that may constitute a disability. Because DLD is known to persist, it would be interesting for future studies to follow up with the DLD only group to examine their parent-reported emotion regulation at a time when we would expect more academic challenge to emerge, such as when comprehension is a greater curricular focus.

Second, our results differ to some extent from studies examining emotion regulation in children with DLD of similar ages to our sample, such that other work has found significant differences between children with DLD and TD children in their emotion regulation (e.g., Burnley et al., 2023; Forrest et al., 2020; Fujiki et al., 2002; Fujiki et al., 2004; Kuusisto et al., 2017; St. Clair et al., 2019; cf. Aguilera et al., 2021; van den Bedem et al., 2018). The number of parents returning BRIEF-2 surveys for children with DLD alone was relatively small; therefore, one possibility is that our study was unable to detect a significant difference between children with DLD alone and children with TD due to reduced power. On the other hand, it is also important to note that there were differences between our study and these previous studies in the method of identification of children with DLD, which could have influenced our results. Some studies enrolled children with previous diagnoses of DLD (Aguilera et al., 2021; Burnley et al., 2023; Fujiki et al., 2002; Fujiki et al., 2004; van den Bedem et al., 2018), and others enrolled children based on parent concerns for language (Forrest et al., 2020; St. Clair et al., 2019). This body of work provides important information about children with DLD who have been clinically identified or for whom parents are aware of their language difficulties, and the findings of these studies likely reflect the caseloads of speech-language pathologists well. However, given the hidden nature of DLD, it can be inferred that many children with DLD in the greater population have not received a formal diagnosis. Further, many parents of children with DLD do not report language concerns (Hendricks et al., 2019). It could be that many of the children with DLD included in the samples of previous studies were identified by parents, teachers, and clinicians as having language difficulties because of co-occurring academic problems (e.g., word reading); should this be the case, our findings partially align with their results, in that we found significantly higher proportions of children with dyslexia+DLD were rated by parents as having elevated emotion regulation difficulties. Our study also included participants without previous diagnoses of DLD, and therefore, our result that children with DLD alone did not significantly differ in their emotion regulation from TD children may reflect the broader DLD profile (though it should be noted that two studies of clinically identified children with DLD also showed null effects: Aguilera et al., 2021 and van den Bedem et al., 2018).

It is important to interpret these results within the context of the current study, which is limited to a single point in time, and to parent reports, which can be subjective. Continued investigations of emotion regulation in children with dyslexia and in children with DLD are needed at multiple stages of development, with careful attention to their co-occurrence; longitudinal work would allow for better understanding of potential causal relationships between emotion regulation and dyslexia and DLD (or vice versa). Parent-report data yields important information about the frequency of emotion regulation difficulties generally displayed by their children under challenge in daily environments. However, these parent ratings reflect broad perceptions of behaviors exhibited by their children, and cannot specify which component of emotion regulation that could be contributing to these behaviors (e.g., emotional reactivity, emotional inhibition, physiological responding, or top-down inhibitory control). Additionally, parents may not witness emotion regulation problems occurring at school or with peers. Future research should employ self-report alongside physiological measures to fully characterize the emotion regulation of children with dyslexia, DLD, and dyslexia+DLD; physiological measures will be especially important given concerns for masking of difficulties in school-age children with DLD (Hobson and Lee, 2023). It would also be useful to examine what abilities may predict the emotion regulation of children within both dyslexia and DLD groups; for example, Aguilera et al. (2021) found that in children with DLD, their expressive vocabulary predicted parent-reported emotion regulation. The role of emotion regulation in development and maintenance of mental health difficulties in children with dyslexia and DLD would be another important direction for examination. For instance, van den Bedem et al. (2018) found that self-reported use of certain emotion regulation strategies like worrying or externalizing predicted depressive symptoms in children with DLD. These further investigations of predictors of emotion regulation and broader mental health difficulties in children with dyslexia and/or DLD could identify important points for intervention to support well-being in these populations.

Another limitation of our study is that due to the design of the larger project, we did not intentionally recruit a separate group of children with ADHD who do not have co-occurring reading or language difficulties, and the number of children with ADHD who were enrolled in the typical language and reading group (*n =* 6) was too small to be considered as its own group for statistical comparisons. Building upon work finding that risk for internalizing and externalizing symptoms is similar in ADHD and ADHD+DLD groups (Hannig Russell and Redmond, 2025), it would be useful for future work to examine whether emotion regulation difficulties present similarly in ADHD and ADHD+dyslexia and ADHD+DLD children. Though in the current study an attenuation of group differences was observed when previous diagnosis of ADHD was considered, this result cannot provide information about whether this attenuation was due to emotional reactivity seen in ADHD (Christiansen et al., 2019) or broader self-regulatory processes associated with ADHD. To begin to answer this question, future studies should examine type and severity of ADHD symptomatology as a predictor of emotion regulation difficulties in children with dyslexia and/or DLD.

## Conclusion

Emotion regulation is an important ability that can support learning and prevent development of mental health problems; until now, research has largely focused on other socioemotional factors in dyslexia, but our study adds information to the literature on a highly treatable factor that can support reading comprehension (Mason et al., 2018), academic success (Graziano et al., 2007), and psychosocial well-being (Beaumont et al., 2023; English et al., 2012; Ricciardi et al., 2022; Seibert et al., 2017). The results of this study show that children with dyslexia may be particularly vulnerable to emotion regulation difficulties, and they align with the growing body of work that has found a significant association between dyslexia and clinical anxiety in children (Donolato et al., 2021; Donolato et al., 2024; Francis et al., 2019; Francis et al., 2022; Grills et al., 2023; McArthur et al., 2022; Robidoux et al., 2025; Vieira et al., 2024). Although ADHD may contribute to emotion regulation problems in children with dyslexia, the present findings suggest that the relationship between dyslexia and emotion regulation difficulties warrants attention in its own right. Our study is the first to consider the co-occurrence of dyslexia and DLD in an examination of emotion regulation in children and provides initial evidence that, at the second-grade level, children with DLD alone do not show increased difficulty in this domain as compared to TD children. One practical implication of our findings is that a significant number of children with dyslexia and dyslexia+DLD may benefit from support to deal with the emotional challenges they experience. When working with children with dyslexia and dyslexia+DLD, teachers should monitor for signs of emotion regulation difficulties, particularly in students who have co-occurring attention or hyperactivity problems. It is also recommended to consult school counselors and psychologists for strategies to support emotion regulation in a classroom or therapeutic environment; providing such support could promote students’ psychosocial well-being and strengthen language and literacy learning. It is vital for future studies to continue to investigate emotion regulation in children with dyslexia, DLD, and dyslexia+DLD to specify the relationships between these factors across childhood. This continued research is essential to clarify developmental pathways and identify transdiagnostic mechanisms linking cognitive, linguistic, and emotional development across childhood and to inform interventions that address both emotional and academic needs.

## Data Availability

The dataset used for analysis is available via OpenScience Framework at https://osf.io/7v9pb.

## Footnotes

1 In response to a reviewer comment inquiring about cohort effects, we also conducted sensitivity analyses to evaluate whether this pattern of results was seen in when evaluating children enrolled in years 1–3 and when evaluating children enrolled in years 4–5. Larger estimates for RD and RD + DLD in comparison to TD were seen in both cohorts; smaller estimates for DLD in comparison to TD were seen in the years 1–3 cohort but in the model for years 4–5, the DLD group would not estimate.
